# Supplementary material for: Neural dynamics of semantic control underlying generative storytelling
Source: Commun Biol. 2025 Mar 28;8:513. doi: 10.1038/s42003-025-07913-3 (PMC11953393; doi:10.1038/s42003-025-07913-3)
Supplement: Supplementary file 2 — Supplementary Material [file 42003_2025_7913_MOESM2_ESM.pdf]

Supplementary information

Word1	Word2	Word3	SET	MSE
libro<br>*(book)*	piazza<br>*(square)*	aereo<br>*(airplane)*	1	0.12374
medico<br>(*physician*)	oca<br>(*goose*)	fiume<br>(*river*)	2	0.12427
grano<br>(wheat)	mago<br>(*magician*)	scuola<br>(*school*)	3	0.12437
pomodoro<br>(*tomato*)	avvocato<br>(*attorney*)	nave<br>(*ship*)	1	0.12455
lattuga<br>(*lettuce*)	attore<br>(*actor*)	moto<br>(*motorcycle*)	2	0.12601
orzo<br>(*barley*)	pilota<br>(*pilot*)	spiaggia<br>(*beach*)	3	0.12481
mela<br>(*apple*)	artista<br>(a*rtist*)	treno<br>(*train*)	1	0.12493
atleta<br>(*athlete*)	foresta<br>(*forest*)	taxi<br>(*taxi*)	2	0.12494
patata<br>(*potato*)	quadro<br>(*painting*)	camion<br>(*truck*)	3	0.12495
limone<br>(*lemon*)	fabbro<br>(*blacksmith*)	canoa<br>(*canoe*)	1	0.12501
cuoco<br>(*chef*)	scarpa<br>(shoe)	parco<br>(*park*)	2	0.12515
fragola<br>(*strawberry*)	maestro<br>(*teacher*)	autobus<br>(*bus*)	3	0.12517
ulivo<br>(*olive tree*)	vigile<br>(*traffic police*)	stereo<br>(*music player*)	1	0.12597
cipolla<br>(*onion*)	delfino<br>(*dolphine*)	ufficio<br>(*office*)	2	0.12607
pastore<br>(*shepherd*)	penna<br>(*pen*)	auto<br>(*car*)	3	0.12614
vaso<br>(*vase*)	aquila<br>(*eagle*)	bici<br>(*bike*)	1	0.12656
soldato<br>(*soldier*)	ape<br>(*bee*)	tram<br>(*tram*)	2	0.12718
pino<br>(*pine*)	orologio<br>(*watch*)	scimmia<br>(*monkey*)	3	0.13194
carota<br>(*carrot*)	Sedia<br>(*chair*)	deserto<br>(*desert*)	1	0.13677
formica<br>(*ant*)	castello<br>(*castle*)	barca<br>(*boat*)	2	0.13704
palla<br>(*ball*)	balena<br>(*whale*)	albergo<br>(*hotel*)	3	0.13753
letto<br>(*bed*)	cane<br>(*dog*)	caverna<br>(*cave*)	1	0.14342
mosca<br>(*fly*)	giardino<br>(*garden*)	pattino<br>(*skate*)	2	0.14406
martello<br>(*hammer*)	gatto<br>*(cat)*	montagna<br>(*mountain*)	3	0.15654

Table S1 Stimuli employed in the Story Generation task (SGT). The first three columns are showing the triplet words for each trial, both in Italian and English. Then, the SET column indicates the three sets of triplets that were counterbalanced across participants. Lastly, the mean square error (MSE) between the pairwise semantic similarity and frequency values of the triplet used to sort and assign them to each set

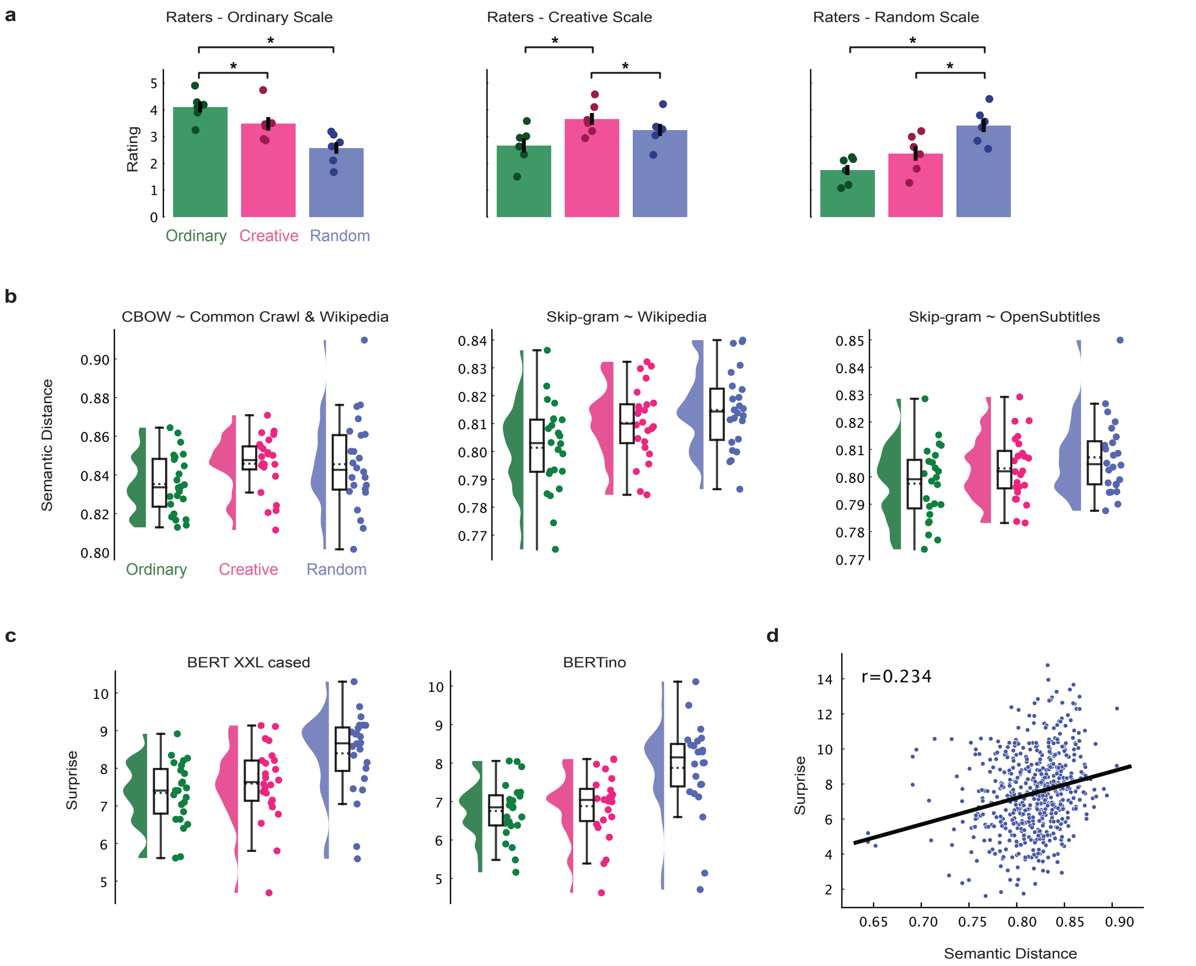

Figure S1 I Behavioural results with human rating, individual models and feature correlation analysis. a. Barplots showing human raters for each scale, with horizontal lines with asterisk indicating statistical significance. Errorbars indicate standard error of the mean (SEM). Raincloud plots depicting the semantic distance in **b** and the surprise scores in **c** for individual models used in the main analysis to show that the results replicate also when considering each model per se. **d.** Scatterplot showing the correlation between the surprise and semantic distance. Black line indicates the regression line fitted to the data.

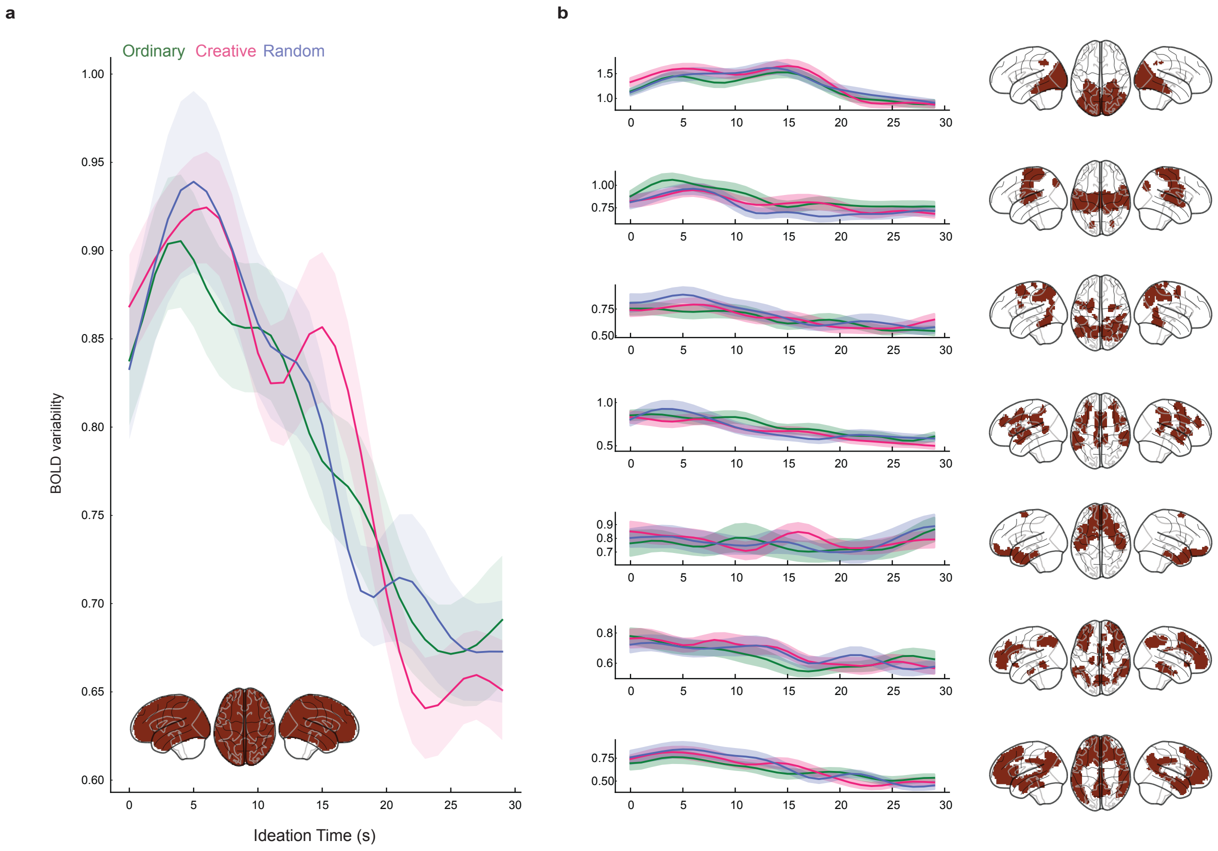

Figure S2 BOLD signal variability as a function of time and condition. Line plots showing the standard deviation of the BOLD signal across trials (stories) at each time point (volume) in the ideation period in the whole brain **(a)** and for each network in the Yeo Atlas **(b)**. Shades indicate standard error of the mean across participants and brain plots highlight the selected areas.

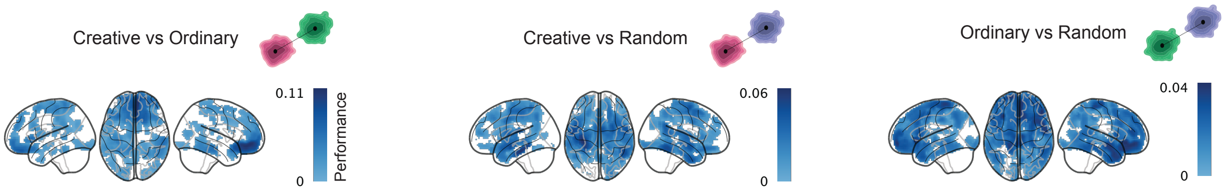

Figure S3 Brain plots showing the searchlight classification results between every pair of conditions, with an alpha threshold for the FDR correction equal to 0.05.

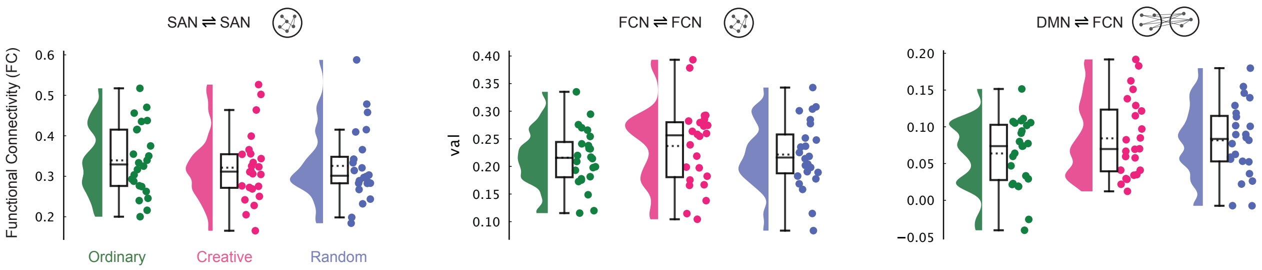

Figure S4 Raincloud plots depicting the functional connectivity values between the brain areas within the SAN (left), the areas within the FCN (center) and the areas between the DMN and the FCN (right).
