# Supplementary material for: Neural dynamics of semantic control underlying generative storytelling
Source: Commun Biol. 2025 Mar 28;8:513. doi: 10.1038/s42003-025-07913-3 (PMC11953393; doi:10.1038/s42003-025-07913-3)
Supplement: Supplementary file 5 — Reporting Summary [file 42003_2025_7913_MOESM5_ESM.pdf]

nature portfolio

Corresponding author(s): Clara Rastelli, Nicola De Pisapia

Last updated by author(s): Mar 4, 2025

Reporting Summary

Nature Portfolio wishes to improve the reproducibility of the work that we publish. This form provides structure for consistency and transparency in reporting. For further information on Nature Portfolio policies, see our Editorial Policies and the Editorial Policy Checklist.

Statistics

For all statistical analyses, confirm that the following items are present in the figure legend, table legend, main text, or Methods section.

n/a	Confirmed	
☐	☒	The exact sample size (*n*) for each experimental group/condition, given as a discrete number and unit of measurement
☐	☒	A statement on whether measurements were taken from distinct samples or whether the same sample was measured repeatedly
☐	☒	The statistical test(s) used AND whether they are one- or two-sided<br>*Only common tests should be described solely by name; describe more complex techniques in the Methods section.*
☐	☒	A description of all covariates tested
☐	☒	A description of any assumptions or corrections, such as tests of normality and adjustment for multiple comparisons
☐	☒	A full description of the statistical parameters including central tendency (e.g. means) or other basic estimates (e.g. regression coefficient) AND variation (e.g. standard deviation) or associated estimates of uncertainty (e.g. confidence intervals)
☐	☒	For null hypothesis testing, the test statistic (e.g. *F*, *t*, *r*) with confidence intervals, effect sizes, degrees of freedom and *P* value noted<br>*Give P values as exact values whenever suitable.*
☒	☐	For Bayesian analysis, information on the choice of priors and Markov chain Monte Carlo settings
☐	☒	For hierarchical and complex designs, identification of the appropriate level for tests and full reporting of outcomes
☐	☒	Estimates of effect sizes (e.g. Cohen's *d*, Pearson's *r*), indicating how they were calculated

Our web collection on statistics for biologists contains articles on many of the points above.

Software and code

Policy information about availability of computer code

Data collection	fMRI data were collected using the acquisition software developed by the system manufacturer
Data analysis	All data analyses were carried out using Python 3.7, the fMRIprep package (23.1.3), the Nilearn library (0.10.3) and the Pytorch library (2.3.1)

For manuscripts utilizing custom algorithms or software that are central to the research but not yet described in published literature, software must be made available to editors and reviewers. We strongly encourage code deposition in a community repository (e.g. GitHub). See the Nature Portfolio guidelines for submitting code & software for further information.

Data

Policy information about availability of data

All manuscripts must include a data availability statement. This statement should provide the following information, where applicable:

- Accession codes, unique identifiers, or web links for publicly available datasets
- A description of any restrictions on data availability
- For clinical datasets or third party data, please ensure that the statement adheres to our policy

Minimally preprocessed behavioural and fMRI data for reproducing the results of this study are openly available at https://osf.io/gxfpc/

Research involving human participants, their data, or biological material

Policy information about studies with human participants or human data. See also policy information about sex, gender (identity/presentation), and sexual orientation and race, ethnicity and racism.

Reporting on sex and gender	Participants were 12 females and 12 males
Reporting on race, ethnicity, or other socially relevant groupings	We randomized the sampling procedure for the ethnicity factor as possible given our local community ethnic distribution.
Population characteristics	Participants were 24 healthy volunteers (12 male) between 19 and 37 years old (mean age 25.67 years, SD = 4.48). None of the volunteers had a history of psychiatric or neurological disor- ders, were on psychoactive drugs, or had any MRI-related contraindications
Recruitment	Participants were recruited via mailing lists at the University of Trento, Italy, as well as from the local community. Some participants were familiar with neuroscience or psychology in general, but not with the specific hypotheses of the study.
Ethics oversight	The study protocol (2020–018) was approved by the Human Research Ethics Com- mittee of the University of Trento. Participants provided their informed consent and were compensated for their participation.

Note that full information on the approval of the study protocol must also be provided in the manuscript.

Field-specific reporting

Please select the one below that is the best fit for your research. If you are not sure, read the appropriate sections before making your selection.

☒ Life sciences ☐ Behavioural & social sciences ☐ Ecological, evolutionary & environmental sciences

For a reference copy of the document with all sections, see nature.com/documents/nr-reporting-summary-flat.pdf

Life sciences study design

All studies must disclose on these points even when the disclosure is negative.

Sample size	The sample size calculation was not based on analytical procedures but on the average sample size of the previous studies in this field.
Data exclusions	Of the 25 volunteers, one was removed because of technical issues during the recording of behavioral data.
Replication	We did not attempt to replicate our findings, as the main result was highly statistically robust.
Randomization	We did not use any randomization as there were no experimental groups.
Blinding	Blinding was not relevant, as there were no experimental groups.

Reporting for specific materials, systems and methods

We require information from authors about some types of materials, experimental systems and methods used in many studies. Here, indicate whether each material, system or method listed is relevant to your study. If you are not sure if a list item applies to your research, read the appropriate section before selecting a response.

Materials & experimental systems

n/a	Involved in the study	
☒	☐	Antibodies
☒	☐	Eukaryotic cell lines
☒	☐	Palaeontology and archaeology
☒	☐	Animals and other organisms
☒	☐	Clinical data
☒	☐	Dual use research of concern
☒	☐	Plants

Methods

n/a	Involved in the study	
☒	☐	ChIP-seq
☒	☐	Flow cytometry
☐	☒	MRI-based neuroimaging

Plants

Seed stocks	*Report on the source of all seed stocks or other plant material used. If applicable, state the seed stock centre and catalogue number. If plant specimens were collected from the field, describe the collection location, date and sampling procedures.*
Novel plant genotypes	*Describe the methods by which all novel plant genotypes were produced. This includes those generated by transgenic approaches, gene editing, chemical/radiation-based mutagenesis and hybridization. For transgenic lines, describe the transformation method, the number of independent lines analyzed and the generation upon which experiments were performed. For gene-edited lines, describe the editor used, the endogenous sequence targeted for editing, the targeting guide RNA sequence (if applicable) and how the editor was applied.*
Authentication	*Describe any authentication procedures for each seed stock used or novel genotype generated. Describe any experiments used to assess the effect of a mutation and, where applicable, how potential secondary effects (e.g. second site T-DNA insertions, mosiacism, off-target gene editing) were examined.*

Magnetic resonance imaging

Experimental design

Design type	Block design
Design specifications	3 blocks of 8 trials each
Behavioral performance measures	Participants were instructed to perform a story generation task (SGT). A set of three target words were presented to participants and they were asked to think at a plot of a story that included these words and then vocalize it. Word triplets were controlled for possible semantic confounds (e.g. semantic similarity, length in letters, avoiding category repetition etc.) and grouped in three sets of 8 word triplets each. The experiment consisted of three conditions where individuals performed the SGT according to different instructions. Participants completed the SGT in three runs of a block design. The order of conditions and the assignment of word triplet sets to each condition were counterbalanced across participants. While the order of triplet presentation within each condition was randomized across participants, the order of words within a triplet remained fixed. Each of the three blocks consisted of 8 trials resulting in a total of 24 stories per participant. Before starting the experiment, in order to familiarize with the task, participants performed 2 practice trials using words that did not appear in the word stimuli used in the experiment. The time-course of a single trial (90 s) is illustrated in Fig. 1d. Each trial began with a fixation-cross in the middle of a black screen as baseline for 25 sec. Then, the word triplet was shown for 5 sec. When the words disappear from the screen, a fixation-cross appeared in the middle of a black screen indicating the beginning of the ideation period, in which participants had to generate the story in their mind for at most 30 seconds. Crucially, during this time period no response was required from participants. Afterwards, the image of a microphone presented on the screen signalled participants to verbally report the generated story. They were instructed to report the story without further elaboration from the ideation period.

Acquisition

Imaging type(s)	functional
Field strength	4T
Sequence & imaging parameters	The scanning duration of each fMRI session was approximately 12 min (730 volumes). fMRI images were acquired with a single shot T2*-weighted gradient-recalled echo-planar imaging (EPI) sequence (TR = 1000 ms, voxel resolution = 3 × 3 × 3 mm3, TE = 28 ms, FA = 59°, FOV = 210 × 210 mm2; slice gap, 0 mm). Moreover, a structural T1- weighted anatomical scan was acquired (MP-RAGE; 1 × 1 × 1 mm3; FOV, 256 × 256 mm2; 176 slices; GRAPPA acquisition with an acceleration factor of 2; TR, 2530 ms; TE1 = 1.64 ms, TE2 = 3.5 ms, TE3 = 5.36 ms, TE4 = 7.22 ms; inversion time (TI), 1100 ms; 7° flip angle).
Area of acquisition	whole brain

Diffusion MRI ☐ Used ☒ Not used

Preprocessing

Preprocessing software	fMRIprep 23.1.3
Normalization	see fMRIprep pipeline
Normalization template	MNI152NLin2009cAsym
Noise and artifact removal	We included in the confounder variables taken from fMRIPrep the six rigid-body motion parameters (three translations and three rotation) and the estimated global, cerebrospinal fluid (CSF) and white matter signal, alongside the first derivatives of all these variables, resulting in a total of 18 variables.
Volume censoring	see fMRIprep pipeline

Statistical modeling & inference

Model type and settings: we performed Multivariate Pattern Analysis (MVPA) on the BOLD signal of the ideation period using the searchlight approach

Effect(s) tested: We classified all the possible pairs of conditions (Creative, Random and Ordinary) at the group-level, by concatenating all the stories from all participants

Specify type of analysis: ☐ Whole brain ☒ ROI-based ☐ Both

Anatomical location(s): Schaefer atlas with 400 ROI

Statistic type for inference (See Eklund et al. 2016): ROI-wise with FDR correction

Correction: FDR

Models & analysis

n/a	Involved in the study
☐	☒ Functional and/or effective connectivity
☒	☐ Graph analysis
☐	☒ Multivariate modeling or predictive analysis

Functional and/or effective connectivity: Gaussian Copula Mutual Information (GCMI)

Multivariate modeling and predictive analysis: we conducted classification analyses using the support vector machine model with the radial basis function as kernel (rbf-SVM) and a regularization value C of 1. Model performance was evaluated using classification accuracy. Statistical analysis were carried out using the 5 × 2 cross-validation F-test. We repeated 5 times a two-fold cross validation (i.e., 50% split) and measured the performance of the model against a model trained with permuted labels for 100 random permutations. Thus, we computed the pseudo f-statistic and the p-value using an F distribution with 10 and 5 degrees of freedom and used the difference between the models' performance as a measure of effect size
